# Specific Activity of Superoxide Dismutase in Stallion Seminal Plasma Is Related to Sperm Cryotolerance

**DOI:** 10.3390/antiox8110539

**Published:** 2019-11-09

**Authors:** Marion Papas, Jaime Catalán, Beatriz Fernandez-Fuertes, Laura Arroyo, Anna Bassols, Jordi Miró, Marc Yeste

**Affiliations:** 1 Equine Reproduction Service, Department of Animal Medicine and Surgery, Faculty of Veterinary Sciences, Autonomous University of Barcelona, E-08193 Bellaterra, Spain; papas.marion@gmail.com (M.P.); dr.jcatalan@gmail.com (J.C.); 2 Biotechnology of Animal and Human Reproduction (TechnoSperm), Unit of Cell Biology, Department of Biology, Institute of Food and Agricultural Technology, Faculty of Sciences, University of Girona, E-17003 Girona, Spain; beatriz.fernandez@udg.edu; 3 Department of Biochemistry and Molecular Biology, Faculty of Veterinary Sciences, Universitat Autònoma de Barcelona, E-08193 Bellaterra, Spain; laura.arroyo88@gmail.com (L.A.); anna.bassols@uab.cat (A.B.)

**Keywords:** seminal plasma, sperm, equine, cryopreservation, antioxidant enzymes

## Abstract

While the removal of seminal plasma is a routine practice prior to equine sperm cryopreservation, this fluid contains the main source of antioxidant enzymes able to scavenge these reactive oxygen species. Therefore, stallion seminal plasma components may have an impact on ejaculate freezability. Against this background, this study was designed to investigate whether the activities of the main stallion seminal plasma antioxidant enzymes are related to sperm cryotolerance. With this purpose, 16 ejaculates were collected from 14 healthy stallions, and each ejaculate was split into two aliquots. The first one was used to evaluate the activities of superoxide dismutase (SOD), catalase (CAT), glutathione peroxidase (GPX), and glutathione reductase (GSR) in seminal plasma. The second aliquot was extended and then processed for cryopreservation. Sperm motility and viability were evaluated before and after cryopreservation, and ejaculates were classified as of good (GFE) or poor freezability (PFE) based on total motile and viable spermatozoa at post-thaw. We observed that, while the specific activities of CAT, GPX, and GSR were similar between GFE and PFE, that of SOD was significantly (*p* < 0.05) higher in GFE than in PFE. We can thus conclude that, in stallions, the specific activity of SOD in the seminal plasma of a given ejaculate might be related to its freezability.

## 1. Introduction

Cryopreserved stallion semen is widely used in the equine breeding industry. Indeed, not only does cryopreservation of stallion sperm facilitate their availability and transport, but it also preserves the genetic material for an unlimited period [1]. However, the plasma membrane of stallion sperm contains an elevated content of polyunsaturated fatty acids [2], which makes this cell highly sensitive to oxidative stress and subsequent lipid peroxidation. Since the process of freezing and thawing exposes sperm to severe cold shock and osmotic stress, their survival and fertilizing ability can be greatly compromised [3]. For this reason, efforts have been made to improve stallion sperm cryopreservation over the last years, testing the use of different cryoprotectants, including glycerol [1]. However, optimizing cryopreservation protocols is still needed, as there is room to increase the fertilizing ability of frozen–thawed stallion sperm. 

While in cattle, selection of sires is based on the assessment of reproductive parameters, including sperm freezability, stallions are selected according to a specific phenotype mainly related to sport performance, which is not usually in line with sperm cryotolerance [1,4]. Moreover, in horses, as in other mammalian species, there is a high individual variability in the ability of sperm to withstand freezing and thawing procedures [4,5,6,7,8]. These differences, which appear to be partially related to the lipid composition of sperm plasma membrane, lead stallions to be classified as of “good” or “poor” freezers [4,6].

Seminal plasma, which is the fluid containing the sperm at ejaculation, is produced by the epididymis and accessory sex glands, and is made up of proteins, ions, and organic substances, such as amino acids, lipids, monosaccharides, and hormones [7,9]. In mammals, this fluid is known to play a vital role for sperm function, both in the male and female reproductive tract [10]; however, conflicting results have been reported with regard to their beneficial or detrimental effects during storage of cooled and cryopreserved equine semen [6,8,9,11]. For this reason, most of the seminal plasma is usually discarded before cryopreservation, as this minimizes the negative impact on sperm motility and viability during sperm storage. However, the inclusion of a low proportion of seminal plasma, from 1% to 20%, has been reported to have a positive effect on sperm motion characteristics [7,8,12].

On the other hand, seminal plasma plays an important antioxidant role against the oxidative damage of spermatozoa. Superoxide dismutase (SOD), catalase (CAT), glutathione peroxidase (GPX), and glutathione reductase (GSR) are the major antioxidant enzymes present in mammalian seminal plasma, and are involved in the scavenging of reactive oxygen species (ROS) [13,14]. As stated above, at the time of ejaculation, sperm and seminal plasma mix. Thus, even in those cases in which seminal plasma is completely removed before cryopreservation, sperm are in contact with seminal plasma for a short period of time. While variations in the composition of seminal plasma have been reported to exist between individual stallions [15,16], no previous study has determined whether the activity of antioxidant enzymes in seminal plasma is related to sperm cryotolerance.

Against this background, the aim of the present work was to determine whether the activities of SOD, CAT, GPX, and GSR in seminal plasma (fresh semen) are related to the sperm ability to withstand the freezing and thawing procedures, as not only could this shed light on how sperm cryopreservation works in equines, but it could also be a useful tool to predict the suitability of a given stallion ejaculate for sperm cryopreservation.

## 2. Materials and Methods 

### 2.1. Experimental Design 

Each ejaculate was split into two fractions. One fraction was centrifuged to recover the seminal plasma and to evaluate the activity of each enzyme, whereas the other was used to evaluate sperm concentration, motility, and viability and then cryopreserved. Upon thawing, sperm motility and viability were also assessed. Based on sperm post-thaw motility and viability, ejaculates were classified as of good (GFE) or poor (PFE) freezability through cluster analyses (see Section 2.9). 

### 2.2. Semen Collection 

Throughout the year, semen samples were collected from 14 warmblood and Arabian stallions, aged from 5 to 21 years old. All animals were housed at the Equine Reproduction Service, Autonomous University of Barcelona (Bellaterra, Cerdanyola del Vallès, Spain). This is an EU-approved semen collection center (Authorization code: ES09RS01E) that operates under strict protocols of animal welfare and health control. All stallions used in this study were semen donors housed at that center and were collected under CEE health conditions (free of Equine Arteritis, Infectious Anemia, and Contagious Metritis). Since this service already runs under the approval of the Regional Government of Catalonia (Spain) and because no manipulation to the animals other than semen collection was carried out, the ethics committee of our institution indicated that no further ethical approval was required. A total of 16 ejaculates (2 from 2 stallions, and 1 from each of the other 12 stallions) was acquired in a regular schedule using a phantom and an artificial vagina Hannover model (Minitüb Ibérica, S.L.; Tarragona, Spain) filled with warm water to obtain a temperature range inside the lumen between 48 °C and 50 °C. An inline nylon filter was used to remove contaminants and the gel fraction. Two equal volume fractions of gel-free semen were obtained from each ejaculate. The first aliquot was used to recover the seminal plasma, whereas the other was diluted 1:4 (*v:v*) with a preheated (37 °C) skim milk extender (4.9% glucose, 2.4% skim milk, 100 mL double-distilled water). This latter aliquot was used to evaluate sperm concentration, viability, and motility in fresh semen, and the remaining volume was intended for cryopreservation.

### 2.3. Seminal Plasma Collection 

Immediately after ejaculate collection, raw semen was placed in 50 mL conical tubes and loaded in a centrifuge at 3000× g and 4 °C for 10 min (JP Selecta S.A., Barcelona, Spain). The supernatant was examined for the presence of sperm cells and centrifuged again and again at the same conditions until seminal plasma was free of spermatozoa. The absence of cells was assessed by a phase-contrast microscope (Olympus Europe, Hamburg, Germany) at 200×. The number of centrifugations depended on the ejaculate, and centrifugation was performed as many times as needed until samples were sperm-free. Usually, around five centrifugations were required. Thereafter, samples were stored in 5 mL tubes at −80 °C until enzyme activities were measured. Before analysis, seminal plasma samples were thawed on ice (4 °C).

### 2.4. Determination of Enzyme Activities 

Tubes containing seminal plasma were thawed prior to evaluating their total protein content and their total and specific activities of the following four antioxidant enzymes: superoxide dismutase (SOD), catalase (CAT), glutathione peroxidase (GPX), and glutathione reductase (GSR).

Total protein content was determined with a biuret-based total protein reagent, which consisted of copper ions in an alkaline reagent that reacted with peptide protein bonds. This reaction resulted in a purple color with a maximum absorbance of 540 nm, directly proportional to the concentration of total protein in the sample. In parallel, a standard curve with bovine serum albumin was prepared. 

Total enzyme activities of SOD, GPX, and GSR in seminal plasma were evaluated using a commercial kit (Randox Laboratories Ltd, Crumlin, UK) following the instructions of the manufacturer. In brief, the assessment of GPX activity (Ransel kit) was performed following the oxidation of the reduced form of nicotinamide adenine dinucleotide phosphate (NADPH) with cumene hydroperoxide and glutathione reductase. Activity of GSR (Glut Red kit) was measured based on the oxidation of NADPH. Finally, the evaluation of SOD activity (Ransod kit) was based on the formation of red formazan dye generated by the reaction of 2-(4-iodophenyl)-3-(4-nitrophenol)-5-phenyltetrazolium chloride (INT) with the superoxide radical produced by xanthine and xanthine oxidase. An Olympus AU400 analyzer (Beckman Coulter, Hamburg, Germany) was used to perform the analytical assessment.

CAT activity was measured through a spectrophotometric assay that monitors the change in absorbance at 240 nm for 30 s, while H_2_O_2_ is reduced to H_2_O and O_2_ [17]. Briefly, 20 µL seminal plasma was diluted in 830 µL phosphate buffer (62.5 mM, pH = 7.2) in a spectrophotometric cuvette. The reaction started following the addition of 150 μl H_2_O_2_ in order to reach a final concentration of 30 mM. Enzyme activity (U/mL) was calculated using the following formula [18]:

$$CAT\;activity\;\left(\frac{U}{L}\right)=\frac{\Delta A}{\varepsilon \times \ell }\;\times \;dilution\;factor$$

where: *A: absorbance*, *ε: extinction molar coefficient (H_2_O_2_)*, ℓ*: light path, dilution factor: 1000*

To evaluate the purity of the enzyme in seminal plasma samples, specific activities of the four enzymes were determined by normalizing the activity of each enzyme with the amount of total protein in seminal plasma samples [19].

### 2.5. Sperm Cryopreservation 

Samples intended for cryopreservation were centrifuged in a Medifriger BL-S programmable refrigerated centrifuge (JP Selecta S.A., Barcelona, Spain) at 600× g and 20 °C for 15 min. Immediately after centrifugation, the supernatant was discarded using a vacuum pump and the sperm pellet was resuspended in a commercial extender containing cryoprotectants (methylformamide and glycerol; BotuCrio, Botupharma, Sweden). Sperm concentration, motility, and viability were again evaluated, and the same commercial extender was added until a final concentration of 200 × 10^6^ viable spermatozoa per mL was reached. Samples were then loaded into 0.5 mL plastic straws and then sealed. Thereafter, straws were cooled and frozen using an Ice-Cube 14S programmable freezer (Minitüb Ibérica, S.L.; Tarragona, Spain) in three steps. First, semen was cooled from 20 °C to 5 °C for 60 min at a rate of −0.25 °C/min. In the second step, the temperature was reduced from 5 °C to −90 °C for 20 min, at a rate of −4.75 °C/min. Finally, the temperature decreased from −90 °C to −120 °C for 2.7 min, at a rate of −11.11 °C/min. Straws were immediately plunged into liquid nitrogen and stored in tanks until thawing and analysis. The thawing protocol consisted of incubating the straws at 37 °C for 30 s in a water bath followed by dilution with three volumes of prewarmed Kenney extender at 37 °C (final concentration: 50 × 10^6^ spermatozoa/mL). After thawing, sperm motility and viability were evaluated at 10 min post-thaw.

### 2.6. Evaluation of Sperm Concentration 

In order to determine sperm concentration, 10 µL of semen was placed in 990 µL buffered saline containing 2% formaldehyde. Three independent counts under a phase-contrast microscope (Olympus 200×; Europe, Hamburg, Germany), using a Neubauer chamber (Paul Marienfeld GmbH & Co. KG; Lauda-Königshofen, Germany), were made.

### 2.7. Evaluation of Sperm Motility 

Sperm motility was evaluated, before and after cryopreservation, using a computer-assisted sperm motility analysis (CASA) system (ISAS 1.0, Proiser; Valencia, Spain) in a prewarmed Neubauer chamber under an Olympus BX41 phase-contrast microscope (Olympus 20× 0.30 PLAN objective; Olympus Europe, Hamburg, Germany) with a heated stage at 37 °C. At least 1000 spermatozoa were counted and the following motility parameters were evaluated: total (TMOT) and progressive sperm motility (PMOT), curvilinear velocity (VCL, µm/s), average path velocity (VAP, µm/s), straight-line velocity (VSL, µm/s), amplitude of lateral head displacement (ALH, µm), beat cross frequency (BCF, Hz), linearity (LIN, %), straightness (STR, %), motility parameter wobble (WOB, %). Settings of the CASA system were: frame frequency = 25 Hz; cell size range = 4–75 µm^2^; connectivity = 12. A spermatozoon was classified as motile when VAP >10 µm/s, and progressively motile when STR ≥75%.

### 2.8. Evaluation of Sperm Viability 

Sperm viability was evaluated before and after cryopreservation through SYBR14 and propidium iodide (PI) using the Live/Dead Sperm Viability kit (Invitrogen Molecular Probes, Thermofisher; Waltham, Massachusetts, USA) and a flow cytometer (Cell Laboratory QuantaSC cytometer, Beckman Coulter; Fullerton, CA, USA). Samples, previously adjusted to 1 × 10^6^ sperm/mL, were stained with SYBR14 (final concentration: 100 nM) at 38.5 °C for 10 min, and with PI (final concentration: 12 µM) at the same temperature for 5 min. Following this, spermatozoa were excited with an argon ion laser emitting at 488 nm and set at a power of 22 mW. The sheath flow rate was set at 4.17 µL/min. Sperm were selected on the basis of electronic volume (EV) and side scatter (SS), and the non-sperm specific events were gated out. The EV channel was periodically calibrated using 10 μm Flow-Check fluorospheres (Beckman Coulter, Brea, California, United States) by positioning this size bead in channel 200 on the EV scale. A total of 10,000 events were evaluated and two optical filters were used: FL1 (green fluorescence): Dichroic/Splitter, DRLP: 550 nm, BP filter: 525 nm; and FL3 (red fluorescence): LP filter: 670/730 nm. Signals were logarithmically amplified and photomultiplier settings were adjusted to each staining method. FL1 was used to detect SYBR14-fluorescence and FL3 was used to detect PI-red fluorescence. Three sperm populations were identified: (i) viable spermatozoa (SYBR14^+^/PI^–^); (ii) non-viable spermatozoa showing red fluorescence (SYBR14^-^/PI^+^); and (iii) non-viable spermatozoa stained both green and red (SYBR14^+^/PI^+^). Non-sperm, debris particles were identified as non-stained either for SYBR14 or PI (SYBR14^−^/PI^−^). SYBR14 spillover into the PI channel was compensated (2.45%). 

### 2.9. Statistical Analyses. 

Data were analyzed with a statistical package (IBM SPSS for Windows Ver. 25.0; IBM Corp., Armonk, NY, USA) and are shown as mean ± standard error of the mean (SEM). First, data were checked for normality (Shapiro–Wilk test) and homoscedasticity (Levene test). Following this, ejaculates were classified into two freezability groups (GFE or PFE) based on their post-thaw sperm viability and total motility, using the likelihood distance and the Schwarz’s Bayesian criterion (cluster analysis). Total and specific enzyme activities between these two groups were compared with a *t*-test for independent samples. Sperm motility and viability before and after freeze–thawing in GFE and PFE were compared through a linear mixed model (intrasubjects factor: before and after cryopreservation; intersubjects factor: GFE v.s. PFE) followed by post hoc Sidak’s test. Pearson coefficient was used to calculate correlations between sperm parameters, and total and specific enzyme activities. The level of significance was set at *p* ≤ 0.05.

## 3. Results

### 3.1. Classification of Ejaculates into Good (GFE) or Poor Freezability (PFE)

According to their post-thaw sperm viability and total motility, stallion ejaculates were classified as of good (GFE) or poor freezability (PFE). From the 16 ejaculates included in this study, 8 were classified as GFE and the other 8 were classified as PFE. Cut-off values for an ejaculate to be considered as GFE were found to be: %TMOT: 52.1%; % viable spermatozoa: 58.6%. Table 1 shows sperm parameters (as mean ± SEM) in fresh and frozen–thawed semen from GFE and PFE. Before cryopreservation, semen quality parameters including viability and progressive and total motilities did not differ between both groups. After thawing, viability, progressive and total motilities, and other kinetic parameters, such as VSL, VAP, LIN, WOB, and ALH, were lower (*p* < 0.05) in PFE than in GFE.

### 3.2. Activity of Superoxide Dismutase in Seminal Plasma of Good and Poor Freezability Ejaculates

**Figure 1:**
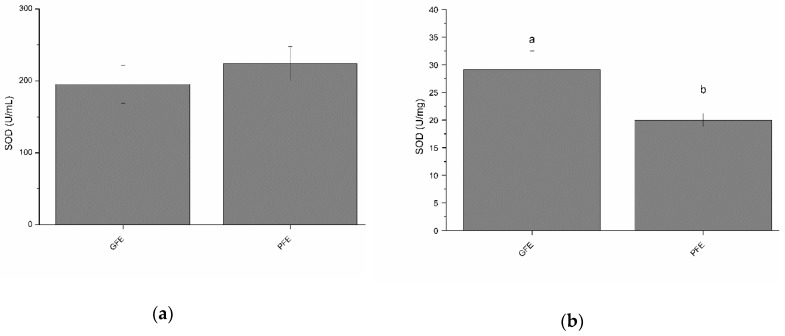
Superoxide dismutase activities in stallion seminal plasma (mean ± SEM): (**a**) total activity; (**b**) specific activity. Different letters (a, b) mean significant (*p* < 0.05) differences between good (GFE) and poor freezability ejaculates (PFE).

Total SOD activity in the seminal plasma of GFE and PFE are shown in Figure 1a (as mean ± SEM). No significant differences were observed between GFE and PFE. However, as shown in Table 2, total SOD activity in seminal plasma negatively correlated (*p* < 0.05) with percentages of progressively motile spermatozoa after thawing. Figure 1b shows the specific SOD activities in GFE and PFE. GFE exhibited higher (*p* < 0.05) specific activity of SOD than PFE. Moreover, SOD specific activities were positively correlated (*p* < 0.05; Table 3) with sperm viability, total sperm motility, VSL, and LIN.

### 3.3. Activity of Catalase in Seminal Plasma of Good and Poor Freezability Ejaculates

**Figure 2:**
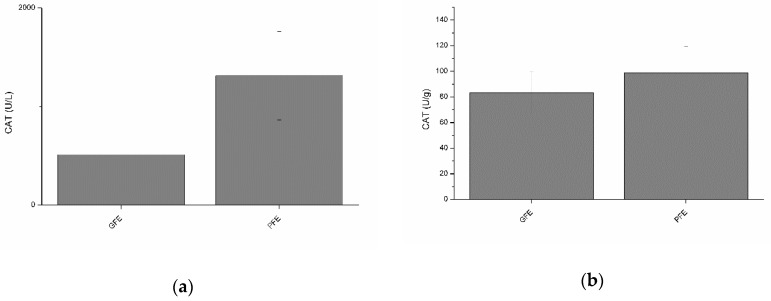
Catalase activities in stallion seminal plasma (mean ± SEM): (**a**) total activity; (**b**) specific activity. No significant (*p* > 0.05) differences between good (GFE) and poor freezability ejaculates (PFE) were observed.

Neither total nor specific activities of CAT were found to differ between GFE and PFE (Figure 2a and b). However, total CAT activity was negatively (*p* < 0.05) correlated with percentages of LIN and STR at post-thaw (Table 2). Moreover, specific CAT activity was negatively correlated (Table 3) with percentages of STR (*p* < 0.01) and BCF (*p* < 0.05) at post-thaw.

### 3.4. Activity of Glutathione Peroxidase in Seminal Plasma of Good and Poor Freezability Ejaculates

**Figure 3:**
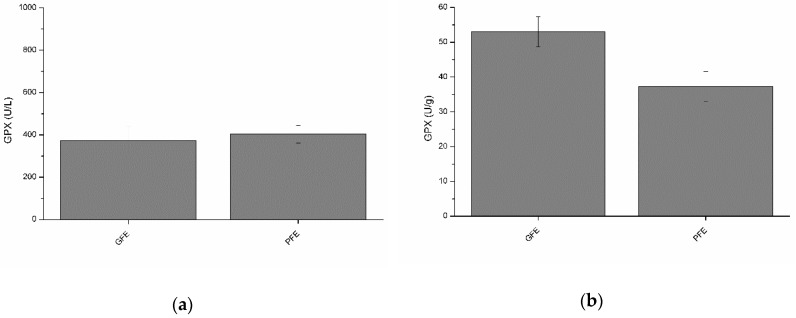
Glutathione peroxidase activities in stallion seminal plasma (mean ± SEM): (**a**) total activity; (**b**) specific activity. No significant (*p* > 0.05) differences between good (GFE) and poor freezability ejaculates (PFE) were observed.

Total and specific activities of GPX are shown in Figure 3a and b, respectively. Although no significant differences between GFE and PFE were observed, total GPX activity was negatively (*p* < 0.05) correlated with post-thaw VCL (Table 2). Moreover, specific GPX activity was negatively (*p* < 0.05) correlated with post-thaw ALH (Table 3).

### 3.5. Activity of Glutathione Reductase in Seminal Plasma of Good and Poor Freezability Ejaculates

**Figure 4:**
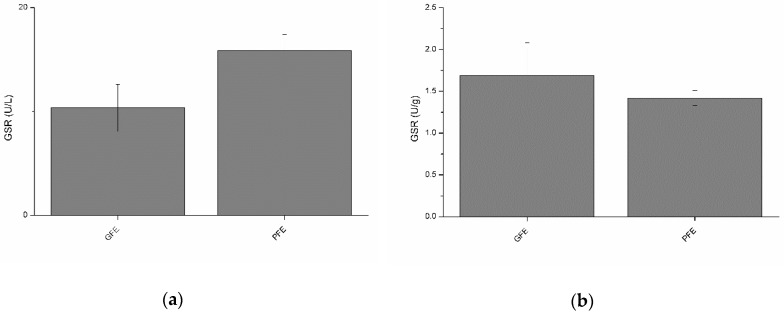
Glutathione reductase activities in stallion seminal plasma (mean ± SEM): (**a**) total activity; (**b**) specific activity. No significant (*p* > 0.05) differences between good (GFE) and poor freezability ejaculates (PFE) were observed.

Total and specific activities of GSR are shown in Figure 4a,b, respectively. No significant differences were observed between GFE and PFE. However, total GSR activity was negatively (*p* < 0.01) correlated with percentages of viable spermatozoa, STR, and BCF at post-thaw (Table 2). Moreover, specific GSR activity (Table 3) was negatively correlated with post-thaw STR (*p* < 0.01) and BCF (*p* < 0.05). Specific GSR activity was also positively correlated with percentages of total motile spermatozoa (*p* < 0.05), VCL (*p* < 0.01), VSL (*p* < 0.01), VAP (*p* < 0.01), and WOB (*p* < 0.01) at post-thaw.

## 4. Discussion

This work was conducted to investigate whether total and specific activities of SOD, CAT, GPX, and GSR in seminal plasma were related to the ability of stallion sperm to withstand cryopreservation. We found that the specific activity of SOD was higher in GFE than in PFE.

Seminal plasma is the fluid, produced by the testis, the epididymis, and the male accessory glands, that accompanies sperm at ejaculation [20]. It provides a suitable environment for sperm, and also represents the most important source of antioxidants, enzymatic and non-enzymatic, able to remove the excess of reactive oxygen species inducing oxidative stress in semen. In that way, removal of seminal plasma prior to cryopreservation may be one of the reasons for the lower fertility of frozen–thawed equine semen [21]. Differences in seminal plasma composition have been observed between and within individuals, and within ejaculates [21]. Equine seminal plasma contains a plethora of enzymes able to scavenge reactive oxygen species, in particular, superoxide dismutase (SOD), catalase (CAT), and glutathione peroxidase/glutathione reductase (GPX/GSR), which are able to catabolize superoxide anion, hydrogen peroxide, and lipid peroxide. The main finding from this study is that, in stallions, the specific SOD activity of seminal plasma is higher in GFE than in PFE. In addition, the specific activity of that enzyme is positively and significantly correlated with post-thaw sperm viability. SOD plays a key role in cell protection against oxidative stress, as it catalyzes the dismutation of superoxide anion into hydrogen peroxide and molecular oxygen [22]. While, in stallion spermatozoa, hydrogen peroxide is usually considered to be the most detrimental ROS, there is, at present, some debate as to whether superoxides are also detrimental [23], since these species do not appear to be converted into hydrogen peroxide [24]. The relationship between SOD activity and sperm function and survival has been already investigated in human and livestock species. Barranco et al. observed that total activity of SOD in boar seminal plasma is positively related with total and progressive sperm motilities and hydrogen peroxide generation by viable spermatozoa, following 72 h of liquid storage at 17 °C [25]. In humans, the reduction of SOD activity in spermatozoa during cryopreservation has been related to lipid peroxidation and subsequent loss of motility after thawing [26]. Moreover, positive correlations have been found between SOD content and the ability of human sperm to withstand freezing and thawing [27]. Based on other studies [28], Buffone et al. assumed a close association between SOD content and SOD enzymatic activity and observed that post-thaw motility recovery was positively correlated with SOD content in mature spermatozoa [27]. Remarkably, in the same study, no significant correlation was found between SOD content in men seminal plasma and motility recovery of spermatozoa after thawing [27], which appears to not match with our results. Under this scenario, the antioxidant power of semen appears to differ between species. Therefore, we suggest that SOD antioxidant protection in human semen is mainly provided by mature spermatozoa rather than by seminal plasma as it occurs in stallions [21]. Moreover, and according to a previous study in stallions, high levels of SOD activity have been found in the ampulla and the prostate, although some activity has also been observed in the other accessory reproductive tissues [21]. Based on our findings, we can hypothesize that stallion semen with higher SOD content in seminal plasma better withstands oxidative stress during cryopreservation. Furthermore, since the activity of this enzyme was evaluated right after ejaculation and seminal plasma was removed prior to cryopreservation, we suggest that a short contact between sperm and seminal plasma proteins is enough to produce a beneficial effect on sperm cryosurvival. Finally, a previous work demonstrated that adding epididymal stallion spermatozoa with seminal plasma may increase the fertility of frozen–thawed semen [29]. Thus, we hypothesize that a quick interaction between sperm cells and seminal fluid is required to increase the sperm resilience to cryopreservation, but a prolonged contact may be detrimental for sperm storage [6]. 

Scavenging activity of SOD is completed by the activity of CAT, which reduces hydrogen peroxide into water and molecular oxygen [22]. In stallions, total and specific activities of CAT in seminal plasma have been shown to be high [14,30], although no correlation has been observed between total activity of CAT in stallion seminal plasma and kinematic sperm parameters [14]. Interestingly, several studies have demonstrated that the addition of CAT to semen prevents the adverse effects of oxidative stress on human [31], mouse [32], boar [33], and stallion [34] spermatozoa. These previous studies support the importance of CAT activity for ROS scavenging and the subsequent modulation of oxidative stress. However, it is worth mentioning that conflicting results have been reported regarding fertility. Indeed, while low total CAT activity has been associated with male infertility in humans [35], the total activity of this antioxidant enzyme has been negatively correlated with the fertility of Arabian horses [36]. Moreover, although previous research supports that total CAT activity in equine semen decreases after freeze–thawing [37], our study has found a negative correlation between total and specific CAT activities and post-thaw kinematic sperm parameters (LIN, STR, and BCF). These differences warrant further research aimed at understanding the importance of the antioxidant system in different mammalian species. 

Protection against ROS-induced damage and maintenance of sperm function and survival are also carried out by the glutathione system (GPX/GSR) [38]. The function of GPX is to reduce hydrogen peroxide to water [22]. Following freeze–thawing, a reduction in the total GPX activity in equine semen has been observed [37]. Furthermore, in Arabian horses, positive correlations of total GPX activity with quality and fertility of fresh equine ejaculate have been observed [36]. In our study, however, total GPX activity was negatively correlated with post-thaw VCL. While these data indicate that the functional role of GPX differs between fresh and frozen–thawed equine spermatozoa, it is worth mentioning that differences between species also exist. Indeed, the relative content of GPX1 and GPX5, which are enzymes that belong to the GPX family, has been found to be positively correlated with sperm cryotolerance, both in humans [38] and in boars [39].

GSR is also associated with antioxidant protection [40] and catalyzes the reduction of glutathione disulfide (GSSG) to the sulfhydryl form of glutathione (GSH). In the present study, we have not observed a clear relationship between total and specific GSR activities and post-thaw sperm quality parameters, which makes the use of this enzyme as a freezability marker difficult. These results match with previous works in humans, in which the relative content of GSR does not appear to be affected during cryopreservation [38]. Although the main finding of this study is that the specific SOD activity in seminal plasma is related to sperm cryotolerance, the other three enzymes investigated (i.e., CAT, GPX, and GSR) were also correlated to some extent negatively or positively to post-thaw sperm motility. In this context, it is worth mentioning that a previous study from our group observed no correlation between SOD, CAT, GPX, and GSR activities in seminal plasma and fresh sperm parameters [38]. In spite of this, the relevance of these four enzymes matches with a previous study from Aurich et al. in which the addition of seminal plasma from GFE was found to improve the sperm resilience to cryopreservation, as it better maintained post-thaw sperm viability and progressive motility [6]. Consequently, the composition of seminal plasma, which appears to differ between GFE and PFE, influences the freezability of stallion spermatozoa. In this context, it is worth remembering that during freezing and thawing, spermatozoa are exposed to changes in osmotic pressure and variations in temperature, which induce oxidative stress and subsequent cryoinjuries [1]. Overproduction of ROS leads to a decrease in survival, acrosome integrity, motility, and fertilizing ability of equine sperm cells [41,42]. In addition, Yeste et al. determined that ROS levels in frozen–thawed stallion spermatozoa differ between GFE and PFE [43]. All these data support the great importance of antioxidant enzymes such as CAT, GPX, GSR, and especially SOD, as its specific activity differs between GFE and PFE, in the correct balance between the radical-generating and radical-scavenging potential. Our results also suggest that the relationship between seminal plasma and the way that sperm cells handle oxidative stress is due, at least in part, to the activity of these four enzymes.

Finally, this study supports the use of specific SOD activity in seminal plasma as a sperm freezability marker in stallions. In this context, it is worth keeping in mind that looking for markers that predict the ability of the ejaculate to be cryopreserved represents a challenge not only in equine but also in other species. In boar seminal plasma, proteins such as non-heparin-binding spermadhesin (PSP-II) or lipocalin enzyme (L-PGDS) have been identified as potential modulators of sperm freezability [44]. In the horse, Bucci et al. observed that the activity of alkaline phosphatase in seminal plasma is positively correlated with post-thaw sperm viability [45]. Herein, we have identified that the specific SOD activity in seminal plasma may also be used as a freezability marker, as it differs between GFE and PFE.

## 5. Conclusions

In summary, the results obtained in this study support the hypothesis that the interaction of seminal plasma components with stallion sperm impacts their freezability. The present study also evidences that the specific activity of SOD in stallion seminal plasma is positively correlated with sperm ability to withstand cryopreservation. Further research to determine whether specific SOD activity varies between ejaculates of the same individual is warranted, since this could address if specific SOD activity in seminal plasma could be useful to classify a given stallion as a ‘good’ or ‘bad’ freezer. 

## Figures and Tables

**Table 1:** Quality parameters (mean ± SEM) of fresh and frozen–thawed semen from good and poor freezability ejaculates (*n* = 16).

	Fresh		Frozen-Thawed	
	GFE	PFE	GFE	PFE
PMOT	45.2 ± 2.9	52.3 ± 1.2	35.1 ± 1.9 **	18.4 ± 1.0 **
TMOT	81.9 ± 3.8	85.6 ± 3.5	58.6 ± 2.1 **	42.0 ± 1.4 **
VCL	102.0 ± 4.1	96.2 ± 4.5	66.3 ± 1.5	63.8 ± 2.4
VSL	53.5 ± 1.7	52.9 ± 2.7	34.8 ± 1.2 *	28.0 ± 1.0 *
VAP	75.1 ± 4.6	67.5 ± 4.5	43.9 ± 2.0 **	35.2 ± 1.3 **
LIN	53.3 ± 3.0	55.0 ± 1.4	52.6 ± 0.7 **	44.0 ± 0.4 **
STR	73.5 ± 4.5	79.2 ± 1.0	79.7 ± 1.1	79.6 ± 0.7
WOB	73.4 ± 2.6	69.5 ± 1.9	66.0 ± 1.5 **	55.2 ± 0.1 **
ALH	2.8 ± 0.2 **	3.6 ± 0.1 **	2.7 ± 0.0 **	3.3 ± 0.1 **
BCF	10.0 ± 0.7 **	12.1 ± 0.5 **	11.7 ± 0.5	12.3 ± 0.3
Viability	79.0 ± 1.3	77.9 ± 1.8	60.0 ± 0.5**	51.35 ± 2.1 **

Abbreviations: GFE: good freezability ejaculates; PFE: poor freezability ejaculates; PMOT: progressive motility; TMOT: total motility; VCL: curvilinear velocity (µm/s); VSL: straight-line velocity (µm/s); VAP: average path velocity (µm/s); LIN: linearity coefficient (%); STR: straightness coefficient (%); WOB: wobble coefficient (%); ALH: lateral head displacement (µm); BCF: frequency of head displacement (Hz). *p < 0.05; **p < 0.01.

**Table 2:** Pearson correlation coefficients between total enzyme activities and post-thawed sperm quality parameters (*n* = 16).

	SOD (U/mL)	GPX (U/L)	GSR (U/L)	CAT (U/L)
PMOT	−0.44 *	−0.04	−0.32	−0.17
TMOT	−0.22	−0.38	−0.09	−0.11
VCL	−0.32	−0.50 *	0.24	−0.11
VSL	−0.33	−0.44	0.02	−0.35
VAP	−0.37	−0.41	0.19	−0.23
LIN	−0.23	−0.22	−0.22	−0.48 *
STR	0.25	0.16	−0.81 **	−0.49 *
WOB	−0.31	−0.25	0.07	−0.29
ALH	0.18	−0.13	0.29	0.44
BCF	0.29	−0.15	−0.62 **	−0.32
Viability	−0.02	−0.30	−0.47 *	0.06

Abbreviations: GFE: good freezability ejaculates; PFE: poor freezability ejaculates; PMOT: progressive motility; TMOT: total motility; VCL: curvilinear velocity (µm/s); VSL: straight-line velocity (µm/s); VAP: average path velocity (µm/s); LIN: linearity coefficient (%); STR: straightness coefficient (%); WOB: wobble coefficient (%); ALH: lateral head displacement (µm); BCF: frequency of head displacement (Hz). *p < 0.05; **p < 0.01.

**Table 3:** Pearson correlation coefficients between specific enzyme activities and post-thawed sperm quality parameters (*n* = 16).

	SOD (U/mg)	GPX (U/g)	GSR (U/g)	CAT (U/g)
PMOT	0.05	0.33	0.38	0.13
TMOT	0.50*	0.08	0.49 *	0.29
VCL	0.18	−0.20	0.60 **	0.27
VSL	0.50 *	0.06	0.66 **	0.11
VAP	0.37	0.03	0.73 **	0.24
LIN	0.62 **	0.26	0.45	−0.12
STR	0.33	0.18	-0.67 **	−0.75 **
WOB	0.45	0.19	0.64 **	0.13
ALH	−0.40	−0.48 *	−0.19	0.26
BCF	0.36	−0.16	−0.50 *	−0.50 *
Viability	0.46 *	0.04	−0.02	0.23

Abbreviations: GFE: good freezability ejaculates; PFE: poor freezability ejaculates; PMOT: progressive motility; TMOT: total motility; VCL: curvilinear velocity (µm/s); VSL: straight-line velocity (µm/s); VAP: average path velocity (µm/s); LIN: linearity coefficient (%); STR: straightness coefficient (%); WOB: wobble coefficient (%); ALH: lateral head displacement (µm); BCF: frequency of head displacement (Hz). *p < 0.05; **p < 0.01.
